# What can the CF registry tell us about rare CFTR-mutations? A Belgian study

**DOI:** 10.1186/s13023-017-0694-1

**Published:** 2017-08-22

**Authors:** E. De Wachter, M. Thomas, S. S. Wanyama, S. Seneca, A. Malfroot

**Affiliations:** 1 CF Clinic, Universitair Ziekenhuis Brussel, Vrije Universiteit Brussel, Laarbeeklaan 101, 1090 Brussels, Belgium; 2 Belgian CF Registry, Scientific Institute of Public Health (WIV-ISP), Brussels, Belgium; 3 Department for Reproduction and Genetics, Centre of Medical Genetics, Universitair Ziekenhuis Brussel, Vrije Universiteit Brussel, Brussels, Belgium

**Keywords:** Cystic fibrosis, CF-registry study, Genotype-phenotype correlation, Disease liability, Rare CFTR-mutations

## Abstract

**Background:**

CFTR2 provides clinical and functional information of the most common CFTR-mutations. Rare mutations (RMs) occur in only a few patients with limited reported clinical data. Their role in CF-disease liability is hardly documented.

**Methods:**

Belgian CF-Registry 2013 data were analyzed to identify CF with at least 1 RM (CF_+RM_). Clinical data and sweat chloride of CF_+RM_ were compared to CF-controls, carrying 2 class 1 to 3 mutations (CF_classic_). Disease severity was compared between both groups. To avoid bias in the comparison, transplanted patients were excluded from each group.

**Results:**

Seventy-seven CF_+RM_ were identified (77/1183 = 6.5%). Sixty-four different RM were detected, of which 21 had not been previously reported. All RMs, corresponding to HGVS (Human Genome Variation Society) nomenclature, were listed in supplementary data.

Seven transplanted CF_+RM_ were excluded for further analysis. CF_+RM_ had higher age at diagnosis [median (IQR)] [3.7 y (0.3–18.3) vs. 0.3y (0.1–2,0) (*p* < 0.0001)], lower sweat chloride [96 mmol/L (64–107) vs. 104 mmol/L (97–115) (*p* < 0.0001)], higher FEV_1_%pred [77%pred (58–96) vs. 68%pred (48–86) (*p* = 0.017)], were less frequently pancreatic insufficient [56% vs. 98% (*p* < 0.0001)], *Pseudomonas aeruginosa* colonized [24% vs. 44% (*p* = 0.0093)] and needed fewer IV antibiotics [36% vs. 51% (*p* = 0.041)] than CF_classic_. However, a wide spectrum of disease severity was seen amongst CF_+RM_.

**Conclusions:**

CF-patients with a RM cover 6.5% of the Belgian CF-population. Rare mutations can be found in severely ill patients, but more often in late diagnosed, pancreatic sufficient patients.

**Electronic supplementary material:**

The online version of this article (doi:10.1186/s13023-017-0694-1) contains supplementary material, which is available to authorized users.

## Background

Cystic fibrosis (CF) is characterized by a wide spectrum of disease severity, ranging from end-stage lung disease at young age to mild symptoms in adulthood. The heterogeneity of mutations on the CFTR-gene is in some way responsible for this finding. However, other genetic modifiers and environmental factors influence disease liability and therefore CFTR-mutations alone should not be used to predict disease severity in CF [1–5].

Since the discovery of the Cystic Fibrosis Transmembrane Regulator (CFTR) gene in 1989 [6], more than 2000 mutations have been detected and reported in the CFTR1-database (CF Mutation Database) [7]. However, not all of these variants are disease-causing. Neutral variants are more frequently seen in the healthy population (*eg*. M470 V, I148T). Some can cause CF in some individuals and no disease in others (*eg*. R117H, D1152H) and are designated as mutations with varying clinical consequences (VCC) [4, 8]. A wide distribution of CFTR variants among different ethnicities and countries has been well described, with F508del being the most prevalent CF-causing mutation worldwide [4, 9].

The aim of CFTR2 (Clinical and Functional Translation of CFTR) was to assess the disease liability of most common CFTR-mutations. Current information in the CFTR2-database is based on phenotypical data from CF-registries of different countries, population studies in healthy subjects and carriers in combination with in vitro functional testing in HeLa and Fisher rat thyroid cells [4, 8]. In August 2015, 276 mutations were listed in CFTR2 and some genotype-phenotype correlations had been reported for most variants [10]. However, the CFTR2-core team warns users of this website database not to use CFTR2 to predict phenotypic outcomes [1–4]. However, CFTR2 may be helpful in diagnostic dilemmas by grouping mutations in 3 different categories (CF-causing, VCC, and Non-CF-causing) [11]. According to the consensus statement, detection of 2 CF-causing mutations in *trans* is one of the criteria to meet in making a diagnosis of CF in individuals with suggestive clinical features [12–14].

Despite attempts to expand CFTR2 to rather rare mutations, little is known about variants that are not listed in CFTR2 and seen in only few people with CF (PWCF) worldwide. No clear definition of a ‘rare CFTR mutation’ (RM) can be found in the literature. We deem that all CFTR mutations not described in CFTR2 could be contemplated as a RM. With the use of extended genotyping, clinicians are more often confronted with the detection of a rare variant of unknown clinical consequence in a patient with suggestive symptoms. Especially in cases of intermediate sweat test values (30–60 mmol/L), CF diagnosis can be hard to make, as this condition can be consistent with CF, CFTR-RD (CFTR-related disorder) or healthy CFTR-carriers. Nasal potential difference (NPD) measurements, intestinal current measurements (ICM) or other in vivo and in vitro functional testing enable a better understanding of the pathogenicity of these mutations and help in refining a diagnosis in these individuals with questionable CF [14–18]. Still, these tests are not easily available and standardized reference data are lacking [19–21]. Results of these functional diagnostic tests are rarely reported to national CF-Registries. However, most of them collect CFTR-genotyping data. Therefore, the CF-registry could be a useful tool to identify CF-patients with uncommon mutations.

We used the Belgian CF-registry database to study CF patients carrying at least 1 RM. Our first aim was to identify all RM and investigate if a RM had been previously reported in CFTR1 or not. Secondly, we compared these patients with age and gender-matched controls with classic CF (carrying 2 class 1–3 mutations) [16, 22], to find out if patients with a RM as a group, differ in disease severity by comparing clinical and sweat test data. Finally, we identified RMs that were seen in patients with sweat chloride values below the diagnostic threshold of 60 mmol/L, as these mutations may be assigned as questionable disease causing mutations.

## Methods

The Belgian CF-registry database of 2013 (BCFR2013) was used for this retrospective study. CF-cases referred to as CF_+RM_, had at least 1 RM defined as a mutation not listed in the CFTR2-database. (Version August 2015) [10]. Some RMs had been previously reported in the CFTR1 database (CFTR1RM) [7], while others had not been previously reported (Non-CFTR1RM).

CF-controls, referred to as CF_classic_, were preferably F508del/F508del, or had another class 1–3 CFTR-mutation on each allele. For the comparison study each CF_+RM_ was age-and gender-matched with 2 CF_classic_. Transplant patients were excluded in each group to avoid bias in comparing respiratory outcomes.

The following variables were analyzed for each study group: age (defined as age on 31st December 2013), age at diagnosis, sweat chloride, FEV_1_%pred, BMI, patients receiving IV antibiotics during 2013, pancreatic status, chronic infection with *Pseudomonas aeruginosa (PA)*, CF- liver disease and CFRD (CF related diabetes). FEV_1_%pred was defined as percentage predicted FEV_1_, using Wang reference values [23] for males between 6 and 17 years and female patients between 6 and 15 years of age, whilst Hankinson reference values were used for males 18 years and above and females 16 years and above [24]. FEV_1_%pred was the result of the last pulmonary function test performed in 2013. BMI was analyzed according to Cachera Z-score, using reference values in males between 0 and 58 years and females between 0 and 56 years [25]. Exocrine pancreatic insufficiency (PI) was defined as fecal elastase < 200 μg/g. Chronic infection with *PA* was defined according to the Leeds criteria [26]. CF-liver disease was considered in cases of cirrhosis with portal hypertension and CFRD if treatment with insulin was required.

‘Missing data’ were data which were not provided by the CF-centre. ‘Not identified’ data were data that could not be demonstrated despite further investigation. Results that seemed to be inconsistent or unclear were double checked with the CF-centre.

All PWCF included in the Belgian CF-Registry signed informed consent to have their data collected and entered into the database as a research tool. Registry data were compliant to Belgian data protection laws.

## Statistics

The data are described using frequencies and proportions, n (%) and mean (standard deviation, SD) or medians (interquartile range, IQR).

To compare the continuous and count data the Mann–Whitney two sample rank test was used where study outcomes failed the normality test. Otherwise, the unpaired Student’s t-test was applied. Differences in cross-tabulated data including the distribution of mutations, CFRD, liver disease, chronic *PA* infection and pancreatic status between both groups were tested using the χ2 statistic. The Fisher’s exact test was used for small counts (≤5), in some cases with continuity correction.

All tests were two sided and considered statistically significant at type I error < 0.05. Where multiple testing was done, the Bonferroni adjustment was used. The analyses were performed using SAS® version 9.3 (SAS Institute Inc.).

## Results

BCFR2013 data included 1186 PWCF, of which 3 had missing genotype data (Table 1). Of the remaining 1183, 995 (84.1%) had a CFTR2-CF-causing mutation on each allele. Nine patients (0.7%) carried at least 1 non-CF-causing variant and 52 patients (4.4%) had at least 1 mutation with VCC, as described by CFTR2 [10]. Fifty patients (4.2%) carried at least 1 mutation that was not identified, of whom 14 (1.2%) had no mutation identified at all.

**Table 1:** BCFR2013-patients, according to their mutations

MUTATION 1	MUTATION 2							
		In CFTR2			Not in CFTR2		Not identified	Total
		CF-causing	Non CF causing	VCC	**CFTR1**	**Non-CFTR1**		
In CFTR2	CF-causing	995	8	49	**51**	**20**	34	1157
	Non CF causing	-	1	-	**-**	**-**	-	1
	VCC	-	-	3	**-**	**-**	-	3
**Not in CFTR2**	**CFTR1**	**-**	**-**	**-**	**2**	**-**	**2** ^**a**^	**4**
	**Non-CFTR1**	**-**	**-**	**-**	**3**	**1**	**-**	**4**
Not identified		-	-	-	**-**	**-**	14	14
Total		995	9	52	**56**	**21**	50	1183

2 patients, carrying 1 RM and 1 ‘not identified’ mutation are excluded from analysis (^a^)

Bold: mutations not reported in CFTR2, subsequently defined as a RM

### Different mutation nomenclature in the registry (see Additional file 1)

As mutations were listed in the way the CF center entered them in the BCFR2013, an attempt was made to reconcile these names with the corresponding HGVS (Human Genome Variation Society) nomenclature (c.DNA and protein name) [27, 28]. Inconsistency in reporting variants lead to confusion in five mutations, incorrectly suggesting these were RM. Consequently, these were removed for further analysis.

### Study population

Seventy-nine CF patients carried at least 1 RM. Two had no second CFTR-mutation identified and were therefore excluded from the study (Table 1). Seventy-seven CF_+RM_ represented 6.5% of the BCFR2013-population. Seven CF_+RM_ underwent lung transplantation and were excluded from the comparison study. For the remaining 70 CF_+RM_, 140 CF_classic_ -non transplanted patients were selected, of which 129 were homozygous F508del and 11 carried 2 class 1–3 mutations on each allele, other than F508del.

#### CF_+RM_ with at least 1 RM documented in CFTR1

Fifty-six CF_+RM_ carried at least 1 CFTR1-documented RM (CFTR1RM) (Table 1). Fifty-one/56 (91%) were compound heterozygous with a CF-causing mutation; 37 carried F508del, 3 had 1717-1G > A, 2 had 2183AA > G and 2 had N1303 K in *trans*, while the following mutations were found once in *trans:* 3272-26A > G, 394delTT, Q890X, 4218insT, G542X, R347P, 2789 + 5G > A.

Two CF_+RM_ carried a CFTR1RM on each allele (1 homozygous, 1 compound heterozygous). Three subjects had in *trans* of their CFTR1RM another RM, which had not previously been listed in the CFTR1 database (Non-CFTR1RM). Altogether 43 different CFTR1RM were found in the BCFR-population (Table 2).

**Table 2:** Rare mutations found in the Belgian CF-population and their prevalence

Prevalence	CFTR1-RM	Non-CFTR1-RM
5×	L165S	
3×	Y913C, M1137R^a^	**1002-1113_110delGAAT**
2×	G458 V, E656X, W882X, 1833delT, **L159S**	Y913S, IVS16-977_IVS17b + 247del2514
1×	E588V, 3272-1G > A, 295ins8, G628R(G > C), 3750delAG, E664X, I105N, 1802delC, 306delTAGA, W1310X, 2116delCTAA, S1455X, P574H, 622-2A > C, 3199del6, c.325 T > C, R258G, c.580-2a > G, 2335delA, Q237E, 1717-3 T > G^b^, W57R, 621 + 2 T > C, 1774delCT, 3600 + 2insT^a^, Q493R^a^, **G213 V** ^c^ **, V562I** ^c^ **, F932S, R810G, P750L, I125T, V938G, c.1076A > G, A120T**	186-2A > G, 3730A > TCT^b^, 2005delTA^c^, K464E, c.1819_1902del, c.1648_1652dupATCAT^c^, CFTRdelePr-1, G85R, c.2909-?_3367 +?del^c^, c.4243-1G > A, p.Gln652x, c.4243_4244insCTGT, c.25dupG, p.Val1240_Gln1291del, c.461dup(p.ala155fs), g.3464_3471dupTCATTGCT;V1198 M, **G551R, T854A**
total	43 mutations	21 mutations

Mutations are written as they are documented in the BCFR2013. For translation into HGVS nomenclature: see Additional file 1

bold: mutations associated with sweat chloride < 60 mmol/L

^a^mutation in *trans* with a Non-CFTR1-RM

^b^homozygous for this RM

^c^mutation in *trans* with a CFTR1-RM

L165S was seen in 5 PWCF, with a CF-causing mutation in *trans*, being the most prevalent RM in Belgium. Based on the registry data, we assumed that 2 patients were siblings and the others are not related.

#### CF_+RM_ with at least 1 RM not documented in CFTR1

Twenty-four CF_+RM_ carried at least 1 RM, not documented in CFTR1 (Non-CFTR1RM) (Table 1). Twenty/24 (83%) were compound heterozygous with a CF-causing mutation, of whom 12 carried F508del in *trans*. The following mutations were seen once in *trans* with the Non-CFTR1RM: 711 + 1G > T, N1303 K, A455E, 360X, R764X, 3849 + 10kbC > T, G542X, 2789 + 5G > A. Three CF_+RM_ carried in *trans* a CFTR1RM, hereby belonging to both groups. (See paragraph 4.2.1) One CF_+RM_ was homozygous for the Non-CFTR1RM. Altogether 21 different Non-CFTR1RM were found in the Belgian CF-population (Table 2).

#### CF_+RM_ with sweat chloride < 60 mmol/L or no sweat test results (Table 3)

Fourteen/77 CF_+RM_ (18%) did not have sweat tests in the CF-range. The lowest sweat chloride (28 mmol/L) was seen in a CF_+RM_ with a RM on both alleles.

**Table 3:** RM with sweat chloride < 60 mmol/L

CFTR1-RM	*Trans* mutation	Sweat Cl (^d^)	Panc	Non-CFTR1-RM	*Trans* mutation	Sweat Cl (^d^)	Panc
G213 V	V562I^a^	28	PI	1002-1113_110delGAAT	F508del	49	PS
F932S	F508del	30	PI	1002-1113_110delGAAT	F508del	38	PS
R810G	F508del	32	PS	1002-1113_110delGAAT	F508del	48	PS
P750L	F508del	36	PI	G551R	3849 + 10kbC > T	30	PS
I125T	F508del	36	PS	T854A	G542X	46	PI
V938G	F508del	40	PS				
Q359R^b^	1717-1G > A	45	PI				
A120T	F508del	47	PI				
L159S^c^	F508del	55	PS				

*Panc* pancreatic status, *PI* pancreatic insufficient, *PS* pancreatic sufficient

^a^CFTR1-RM

^b^Q359R = legacy name for c.1076A > G, as mentioned in the registry

^c^Found in another subject with sweat chloride > 60 mmol/L

^d^in mmol/L

Seven adults with CFTR1RM had no sweat test data (missing data). All had an early diagnosis (before the age of 2 years) and were mainly pancreatic insufficient (PI): 5/7 PI, 1/7 PS and 1/7 missing data.

#### CF_+RM_ cases with lung transplantation (Table 4)

Seven CF_+RM_ underwent lung transplantation. All carried F508del in *trans* of their RM. Importantly, age at diagnosis differed in this group (range 0–13 years). They all had clear abnormal sweat tests (1 missing) and were mostly PI. This group was excluded for comparison between CF_+RM_ and CF_classic_.

**Table 4:** CF_+RM_ with lung transplantation

CFTR1-RM	CFTR-RM	*Trans* mutation	Age at transplant	Sweat chloride (mmol/L)	Pancreatic Status
Y	E656X	F508del	25-30y	124	PS
Y	Y913C	F508del	30-35y	118	PS
Y	3750delAG	F508del	25-30y	112	PI
Y	E664X	F508del	30-35y	Missing	PI
Y	M1137R	F508del	25-30y	101	PI
Y	1833delT	F508del	30-35y	115	Missing
N	IVS16-977_IVS17b + 247del2514	F508del	20-25y	115	PI

*PI* pancreatic insufficient, *PS* pancreatic sufficient

### Comparison of CF_+RM_ with CF_classic_ (Table 5)

Each CF_+RM_ (*n* = 70) was matched for age and gender with 2 CF_classic_ (*n* = 140).

**Table 5:** Comparison of CF_+RM_ with age and gender controlled CF_classic_

CCharacteristics	CF_+RM_	CF_classic_	*p*-value
Number (n)	70	140	
Male	37 (52.9%)	74 (52.9%)	1.0000
Adults	46 (65.7%)	88 (62.9%)	0.6846
Mutation-distribution			
F508del homozygous	0 (0.0%)	129 (92.1%)	-
F508del heterozygous	42 (60.0%)	0 (0.0%)	**-**
other	28 (40.0%)	11 (7.9%)	**< 0.0001**
Age (y)	27.6 (11.9–37.3)	26.1 (13.2–33.7)	
	25.9 (15.0)	24.3 (13.3)	0.5406
Age at diagnosis (y)	70 (100%)	140 (100%)	
	3.7 (0.3–18.3)	0.3 (0.1–2.0)	**< 0.0001**
Sweat chloride^Ȣ^ (mmol/L)	64 (91,4%)	117 (83.6%)	
	96.3 (63.7–107.0)	104.0 (97.1–115.0)	**< 0.0001**
Sweat chloride^Ȣ^			
Missing	6 (8,6%)	23 (16.4%)	0.1198
0- ≤ 30 mmol/L	3 (4.3%)	0 (0%)	0.0360^**ȸ**^
31- ≤ 60 mmol/L	11 (15,7%)	0 (0%)	< 0.0001 ^**ȸ**^
> 60 mmol/L	50 (71,4%)	117 (83.6%)	**0.0398**
FEV_1_%pred	59 (84.3%)	123 (87.9%)	
	76.7 (57.8–95.9)	68.4 (48.1–85.6)	0.0166*
	76.8 (25.4)	67.4 (24.1)	
BMI-Z score (Cachera)	65 (92.9%)	145 (97.9%)	
	− 0.3 (− 1.0–0.4)	− 0.5 (− 1.1–0.1)	0.2059
Patients with IV AB	25 (35.7%)	71 (50.7%)	**0.0414**
Days of IV AB^a^	21.0 (11.0–48.0)	18.0 (14.0–42.0)	0.5690
Pancreatic insufficiency	39 (55.7%)	137 (97.9%)	**< 0.0001**
Chronic *PA*	17 (24.3%)	61 (43.6%)	**0.0093**
CF liver disease	3 (4.3%)	8 (5.7%)	0.7551**
CFRD	14 (20.0%)	32 (22.9%)	0.8432

Data are presented in n (%), Median (IQR) or mean (SD)

*p*-value: statistical significant if < 0.05 bold

* Two sample t-test

** Fisher’s exact test

^**ȸ**^ Fisher’s exact test with continuity correction

^**Ȣ**^Bonferroni adjusted threshold *P* < 0.01

^**a**^Days of IV AB (antibiotics): in patients who received IV antibiotics

Age at diagnosis was statistically higher in CF_+RM_ compared to CF_classic_ (*p* < 0.0001). CF_+RM_ had lower sweat chloride levels than CF_classic_ (*p* < 0.0001). All CF_classic,_ for whom sweat test data were available, had a sweat chloride > 60 mmol/L. This was only the case in 79% of CF_+RM._. Compared to CF_classic_, this is a statistically significant difference (*p* < 0.0001). CF_+RM_ had better FEV1%pred compared to CF_classic_ (*p* = 0.0166) and were less frequently *PA*-colonized than CF_classic_ (*p* = 0.0093). CF_+RM_ were less likely to receive IV antibiotics than CF_classic_ (*p* = 0.0414) and were less frequently PI (*p* < 0.0001). No difference could be seen on BMI z-score, CF-liver disease or CFRD between the two groups.

## Discussion

The BCFR2013 revealed that 84.1% of the Belgian CF patients could have been diagnosed based only on genetic testing, using sequencing and CFTR2-criteria. However, other non-genetic diagnostic tests (sweat test, NPD, ICM) are needed to confirm a CF-diagnosis in patients with at least 1 mutation with VCC (4.4%), patients who have no second mutation identified (4.2%) and those who carry at least 1 RM (6.5%). This is consistent with the findings of Ooi et al. who demonstrated that the use of mutations as a diagnostic tool is of limited value compared to functional testing such as sweat testing and NPD, especially in people with mild clinical presentation [29, 30]. In 4.4% of the BCFR-population, at least 1 CFTR-mutation is missing or unknown. Some of these PWCF may carry a rare, not yet identified CFTR-mutation. Extended sequencing analysis of the whole CFTR gene should be proposed in these individuals.

In this study 64 different RM were detected, of which 21 had not been previously reported. Belgium contributes data to CFTR1 and CFTR2. However, we should encourage CF-centres to continue reporting their rare mutations to CFTR1 to overcome underreporting in the future. To our knowledge, L165S, the most prevalent RM in Belgium, has only been described in CFTR1 in 2 French adults [7].

CF patients with at least 1 RM, as a group, had significantly more preserved pancreatic and pulmonary function than their CF-controls, carrying 2 class 1–3 mutations, explaining the significant differences in sweat chloride, the later diagnosis and the reduced need for IV antibiotic treatment in CF_+RM_ compared to CF_classic_. However, it cannot be concluded that patients with a RM always have milder disease. In this group, 7 CF_+RM_ underwent lung transplantation. Moreover, the 64 detected RM comprise frameshift mutations, PTC mutations and large deletions, suggesting importantly disrupted and non-functional CFTR. As for most common CFTR-mutations, a broad range in severity of RM is seen and this is responsible for the wide range in phenotype in these individuals [1, 2]. In the case of compound heterozygosity, the mildest mutation is known to be the most dominant on phenotype [1, 2, 10].

Eighteen percent of the CF_+RM_ had a sweat chloride < 60 mmol/L. Further functional testing in these patients should be proposed in order to find out if both mutations are CF-causing and if CF-diagnosis in these individuals could be demonstrated. Data from the literature and CFTR1 suggest that most of the RMs found in our CF_+RM_ with a sweat chloride < 60 mmol/L are related to CFTR-RD or are seen in asymptomatic subjects [7]. (See Additional file 1). However, based on registry data alone we will not be able to predict disease liability of a RM. Collecting clinical data and measuring CFTR-function in vivo and ex vivo in a prospective way in a large population will be the only way to get a better understanding of the pathogenicity of RMs. This strategy is the goal of the CFTR3-project [31].

CF registries are of valuable use in studying CF populations in general and in comparing groups and trends in variables over time [32, 33]. However, the use of registry data has its limitations. Entering patients’ data into a CF-registry does not necessarily imply that this person is affected with CF. Thomas et al. showed that not all registry patients fulfil predefined CF diagnostic criteria [34]. To get more accurate information in case of inconsistency, it is preferable to contact the CF-centre who entered these data, which was done accordingly in our study. Despite these efforts, some inconsistencies (ie. PI in patients with borderline sweat tests, typographical errors) remained unchanged. Previous publications have highlighted the problem of data quality in CF registries as a limitation of studies based on registries [1–3, 27, 34].

An important impediment we faced was the mixture of different nomenclatures for CFTR-mutations used in the BCFR2013. Berwouts and co-workers have shown that completing data in a consistent way by the molecular lab should be done when reporting a CFTR-variant. However, this is hardly the case and may result in misinterpretation [27]. Genetic labs should be aware of this and reports of CFTR-analysis should contain all information that is needed to avoid confusion [28]. Furthermore, CF-registries are mostly entered by CF-team members, who are not familiar with HGVS-nomenclature. Misspelling of a mutation may also lead to errors and should be regularly checked.

Inconsistency in reporting variants lead to confusion in 5 of our cases. 2184AA > G and 2181AA > G were initially considered as a RM, as no match with a CFTR2, nor a CFTR1 variant was found. However, both are an alternative (not commonly accepted) description of 2183AA > G, being a CFTR2-mutation. The second ambiguous name was c.[1680-886A > G], better known as the old nomenclature name c.1679 + 1.6kbA > G (legacy name 1811 + 1.6kbA > G), being a CFTR2 mutation and earlier described by Chillon [35]. An alternative, but not commonly accepted nomination of IVS16-977_IVS17b + 247del2514 was del exon 17. The four alternative nomenclatures we found were not the consequence of a misspelling at registry level. Entering an ENaC mutation as if it was a CFTR-mutation in the BCFR-2013 lead again to confusion. CF-registries do not provide the possibility to enter mutations at another level such as CFTR. Moreover, segregation analysis is needed to confirm the location of mutations in *trans.* This should always be stated in the final molecular report to avoid misdiagnosis [2, 36]. To overcome these limitations in the future, reporting CFTR-variants in CF-registries should be done in a meticulously way; avoiding typographical errors, entering c.DNA name, protein name and legacy name, if available, and confirming that both mutations are located in *trans*. This study shows that regular reviews by a geneticist in the CF-field would contribute to better reporting of RM in CF-registries and should therefore be encouraged.

## Conclusion

This is the first national CF-registry study where data about RMs are collected and compared to classic CF-controls. Whether a mutation could be assigned as a RM depends on the population and the timeframe in which the mutation is found. CFTR2 is continuously expanding. Mutations that are currently considered as a RM can become more common in the future because of a better reporting worldwide. We were able to identify 64 RMs in Belgium of which an important percentage had never been reported before. CF patients carrying at least one RM are more likely to have milder disease than classic CF patients. However, a wide range in disease severity is seen. Based on registry data alone, we will not be able to define the disease liability of a RM. Therefore, a prospective study, using electrophysiological tests in subjects with the identified RMs will be needed.

We can conclude that this survey is a first step in identifying CF-patients with RMs in a prospective way. Currently, patients with RMs do not have access to new CFTR-modulators nor can they participate in running clinical trials. Documenting the effect of the RM on CFTR-protein function will enable better classification of the RM with the aim to get access to personalized therapy in the future.

## Additional file

Additional file 1: Table S1. CFTR mutations reported in the BCFR2013: translation into HGVS nomenclature (NM_000492.3) and additional information for CFTR1-RM with sweat chloride < 60 mmol/L. (DOCX 29 kb)

## Abbreviations

BCFR2013: Belgian CF-Registry of 2013
CF_+RM_: CF patient with at least 1 RM
CF_classic_: CF patient with 2 class 1–3 mutations (classic CF)
HGVS: Human Genome Variation Society
Non-CFTR1RM: Rare mutations that have never been reported in the CFTR1-database until now (July2016)
PWCF: people with Cystic Fibrosis
RM: Rare mutation
VCC: Varying clinical consequence
