# Supplementary material for: What can the CF registry tell us about rare CFTR-mutations? A Belgian study
Source: Orphanet J Rare Dis. 2017 Aug 22;12:142. doi: 10.1186/s13023-017-0694-1 (PMC5567473; doi:10.1186/s13023-017-0694-1)
Supplement: Additional file 1: Table S1. — CFTR mutations reported in the BCFR2013: translation into HGVS nomenclature (NM_000492.3) and additional information for CFTR1-RM with sweat chloride < 60 mmol/L. (DOCX 29 kb) [file 13023_2017_694_MOESM1_ESM.docx]

**Additional file 1**

**Table S1: CFTR mutations reported in the BCFR2013**: translation into HGVS nomenclature (NM_000492.3) and additional information for CFTR1-RM with sweat chloride <60 mmol/L

| **RM as reported in the BCFR2013**  **CFTR1-RM** | **Legacy name** | **c.DNA name** | **Protein name** | **Preval.** | **Remark and/or**  **Comment in CFTR1^†^ [1]** |
| --- | --- | --- | --- | --- | --- |
| L165S | L165S | c.[494T>C] | p.(Leu165Ser) | 5x |  |
| Y913C | Y913C | c.[2738A>G] | p.(Tyr913Cys) | 3 |  |
| M1137R | M1137R | c.[3410T>G] | p.(Met1137Arg) | 3 |  |
| G458V | G458V | c.[1373G>T] | p.(Gly458Val) | 2 |  |
| E656X | E656X | c.[1966G>T] | p.(Glu656*) | 2 |  |
| W882X | W882X | c.[2645G>A] | p.(Trp882*) | 2 |  |
| 1833delT | 1833delT | c.[1703delT] | p.(Leu568Cysfs*4) | 2 |  |
| L159S | L159S | c.[476T>C] | p.(Leu159Ser) | 2 | Once found with sweat chloride <60mmol/L |
| E588V | E588V | c.[1763A>T] | p.(Glu588Val) | 1 |  |
| 3272-1G>A | 3272-1G>A | c.[3140-1G>A] | p.? | 1 | Intronic mutation |
| 295ins8 | 295ins8 | c.[163_164ins8] | p.(Arg55Asnfs*39) | 1 |  |
| G628R(G>C) | G628R(G>C) | c.[1882G>C] | p.(Gly628Arg) | 1 |  |
| 3750delAG | 3750delAG | c.[3618_3619delAG] | p.(Gly1208Profs*56) | 1 |  |
| E664X | E664X | c.[1990G>T] | p.(Glu664X) | 1 |  |
| I105N | I105N | c.[314T>A] | p.(Ile105Asn) | 1 |  |
| 1802delC | 1802delC | c.[1670delC] | p.(Ser557Phefs*2) | 1 |  |
| 306delTAGA | 306delTAGA | c.[174_177delTAGA] | p.(Asp58GlufsX32) | 1 |  |
| W1310X | W1310X | c.[3929G>A] | p.(Trp1310X) | 1 |  |
| 2116delCTAA | 2116delCTAA | c.[1984_1987delCTAA] | p.(Thr663Argfs*8) | 1 |  |
| S1455X | S1455X | c.[4364C>G] | p.(Ser1455*) | 1 |  |
| P574H | P574H | c.[1721C>A] | p.(Pro574His) | 1 |  |
| 622-2A>C | 622-2A>C | c.[490-2A>C] | p.? | 1 | Intronic mutation |
| **RM as reported in the BCFR2013**  **CFTR1-RM** | **Legacy name** | **c.DNA name** | **Protein name** | **Preval.** | **Remark and/or**  **Comment in CFTR1^†^ [1]** |
| 3199del6 | 3199del6 | c.[3067_3072delATAGTG] | p.(Ile1023_Val1024del) | 1 |  |
| c.325T>C | c.325T>C | c.[325T>C] | p.(Tyr109His) | 1 |  |
| R258G | R258G | c.[772A>G] | p.(Arg258Gly) | 1 |  |
| c.580-2a>G | 712-2A>G | c.[580-2A>G] | p.? | 1 | Intronic mutation  'a' should be changed into 'A' |
| 2335delA | 2335delA | c.[2203delA] | p.(Arg735Glyfs*4) | 1 |  |
| Q237E | Q237E | c.[709C>G] | p.(Gln237Glu) | 1 |  |
| 1717-3T>G | 1717-3T>G | c.[1585-3T>G] | P? | 1 | Intronic mutation |
| W57R | W57R | c.[169T>C] | p.(Trp57Arg) | 1 |  |
| 621+2T>C | 621+2T>C | c.[489+2T>C] | P? | 1 | Intronic mutation |
| 1774delCT | 1774delCT | c.[1642_1643delCT] | p.(Leu548Glufs*19) | 1 |  |
| 3600+2insT | 3600+2insT | c.[3468+2_3468+3insT] | P? | 1 | Intronic mutation |
| Q493R | Q493R | c.[1478A>G] | p.(Gln493Arg) | 1 |  |
| **G213V** | G213V | c.[638G>T] | p.(Gly213Val) | 1 | *CFTR1:* identified in a CF carrier |
| **V562I** | V562I | c.[1684G>A] | p.(Val562Ile) | 1 | Discrepancy in literature about disease liability:  *- CFTR1*: 1 case homozygous with severe CF and PI  - *CFTR1*: Fanen (1992) reported as a polymorphism  *- [2]*: not disease causing |
| **F932S** | F932S | c.[2795T>C] | p.(Phe932Ser) | 1 | *CFTR1*: 1 asymptomatic subject |
| **R810G** | R810G | c.[2428A>G] | p.(Arg810Gly) | 1 | *CFTR1:* 1 CBAVD (F508del in *trans*) |
| **RM as reported in the BCFR2013**  **CFTR1-RM** | **Legacy name** | **c.DNA name** | **Protein name** | **Preval.** | **Remark and/or**  **Comment in CFTR1^†^ [1]** |
| **P750L** | P750L | c.[2249C>T] | p.(Pro750Leu) | 1 | *CFTR1* describes 3 other subjects: -1 of 2m, PI and lung disease  -1 of 8y with asthma and sweat test of 90 mmol/L  -1 with asthma and PI |
| **I125T** | I125T | c.[374T>C] | p.(Ile125Thr) | 1 | *CFTR1:* 1x Chinese women, no additional info  In literature described as CFTR-Related Disorder (CFTR-RD):  - Described in Asian population with recurrent pancreatitis [3]  - described in Idiopathic bronchiectasis [4] |
| **V938G** | V938G | c.[2813T>G] | p.(Val938Gly) | 1 | *- CFTR1:* Seen in 2 men:  1 homozygous: CUAVD, PS and asthma  1 heterozygous with a frameshift mutation: CBAVD  - 1 case of CUAVD [5] |
| **c.1076A>G** | Q359R | c.[1076A>G] | p.(Gln359Arg) | 1 | *CFTR1*: 1 asymptomatic subject |
| **A120T** | A120T | c.[358G>A] | p.(Ala120Thr) | 1 | *- CFTR1*: 1 subject with 5T in *Trans* and sweat test of 90 mmol/L  - 1 boy, lung disease 1^st^ months of life, twice sweat test <30 mmol/L [6] |
| **RM as reported in the BCFR2013**  **Non-CFTR1-RM** | **Legacy name** | **c.DNA name** | **Protein name** | **Preval.** | **Remark and/or**  **Comment in CFTR1^†^ [1]** |
| **1002-1113_110delGAAT** |  |  |  | 3 | Not interpretable: typographical error? However, 3 times present in BCFR2013. |
| Y913S |  | c.[2738A>C] | p.(Tyr913Ser) | 2 |  |
| c.[1680-886A>G] | 1811+1.6kbA>G | c.[1680-886A>G] | p.? |  | The old nomenclature is c.1679+1.6kbA>G and its legacy name 1811+1.6kbA>G, which is a common Spanish mutation and known in CFTR2. [7] [communication by J Rommens (Toronto)]  Therefore this mutation was excluded from the analysis |
| 186-2A>G |  | c.[52-2A>G] | p.? | 1 | Intronic mutation |
| 2184AA>G | 2183AA>G | c.[2051_2052delAAinsG] | p.(Lys684Serfs*38) |  | Different description of a common (CFTR2) mutation: 2183AA>G. Therefore this mutation is excluded from analysis |
| 3730A>TCT |  | c.[3598delinsTCT] | p.(Lys1200Serfs*12) | 1 |  |
| IVS16-977_IVS17b+247del2514 | IVS16-977_IVS17b+247del2514 | c.[2909-977_3367+247del] | p.? | 2 | Intronic mutation |
| **RM as reported in the BCFR2013**  **Non-CFTR1-RM** | **Legacy name** | **c.DNA name** | **Protein name** | **Preval.** | **Remark and/or**  **Comment in CFTR1^†^ [1]** |
| del exon 17 | IVS16-977_IVS17b+247del2514 |  |  |  | Del exon 17, was later reported by the molecular lab as IVS16-977_IVS17b+247del2514, being the same as abovementioned variant |
| 2005delTA |  | c.[1874_1875del] | p.(Tyr625Phefs*16) | 1 |  |
| K464E |  | c.[1390A>G] | p.(Lys464Glu) | 1 |  |
| c.1819_1902del |  | c.[1819_1902del] | p.(Met607_Gln634del) | 1 |  |
| L180L-R181W |  |  |  |  | This is no CFTR-mutation but an ENaC mutation |
| c.1648_1652dupATCAT |  | c.[1516_1520dup] | p.(Phe508Serfs*21) | 1 |  |
| CFTRdelePr-1 |  |  |  | 1 | Not interpretable |
| G85R |  | c.[253G>A] | p.(Gly85Arg) | 1 |  |
| c.2909-?_3367+?del |  | c.[2909-?_3367+?del] | p.? | 1 | Intronic mutation |
| 2181AA>G | 2183AA>G | c.[2051_2052delAAinsG] | p.(Lys684Serfs*38) |  | Different description of a common (CFTR2) mutation: 2183AA>G. Therefore this mutation is excluded from analysis |
| c.4243-1G>A |  | c.[4243-1G>A] | p.? | 1 | Intronic mutation |
| p.Gln652x |  | c.[1954C>T] | p.(Gln652*) | 1 |  |
| c.4243_4244insCTGT |  | c.[4243_4244insCTGT] | p.(Val1415Alafs*48) | 1 |  |
| c.25dupG |  | c.[25dup] | p.(Ala9Glyfs*36) | 1 |  |
| p.Val1240_Gln1291del |  | c.[3718?_3873+?del] | p.(Val1240_Gln1291de)l | 1 |  |
| c.461dup(p.ala155fs) |  | c.[461dup] | p.(Ala155Serfs*4) | 1 |  |
| g.3464_3471dupTCATTGCT;V1198M |  | c.[3332_3339dup;3592G>A] | p.(Val1114Serfs*10) | 1 | Complex allele |
| **G551R** |  | c.[1651G>C] | p.(Gly551Arg) | 1 |  |
| **T854A** |  | c.[2560A>G] | p.(Thr854Ala) | 1 |  |

**^†^** Only done for CFTR1-RM with sweat chloride <60 mmol/L
<**bold**>: mutations with sweat chloride <60 mmol/L
Grey background: 5 reported mutations, incorrectly suggesting these were RM
Preval.: prevalence in the BCFR2013
CUAVD: Congenital unilateral absence of the vas deferens

**References:**

1. CFTR1 website: <http://www.genet.sickkids.on.ca-> version June 2016; last update 25 April 2011.

2. Roxo-Rosa M, Xu Z, Schmidt A, Neto M, Cai Z, Soares CM et al. Revertant mutants G550E and 4RK rescue cystic fibrosis mutants in the first nucleotide-binding domain of CFTR by different mechanisms. PNAS 2006;103:17891-17896. [www.pnas.org/cgi/doi/10.1073/pnas.0608312103](http://www.pnas.org/cgi/doi/10.1073/pnas.0608312103).

3. Chang, MC, Jan IS, Liang PC, Jeng YM, Yang CY, Tien YW et al. Cystic fibrosis transmembrane conductance regulator gene variants are associated with autoimmune pancreatitis and slow response to steroid treatment. J Cyst Fibros 2015; 14(5):661-667. DOI:10.1016/j.jcf.201503009.

4. Ngiam NSP, Chong SS, Shek LPC, Goh DLM, Ong KC, Cheng SY et al. Cystic fibrosis transmembrane conductance regulator (CFTR) gene mutations in Asians with chronic pulmonary disease: A pilot study. J Cyst Fibros 2006;5:159-164. Doi: 10.1016/j.jcf.2006.02.002.

5. RobinG, Lefèbvre-Khalil V, Dumur V, Lemaître L, Mitchell V, Rigot JM et al. Unilateral partial deferential agenesia and CFTR gene composite heterozygoty (Delta F508/V938G). Gynécologie obstétrique et fertilité 2007;35:561-564.

6. Padoan R, Genoni S, Moretti E, Seia M, Giunta A, Corbetta C. Genetic and clinical features of false-negative infants in a neonatal screening program for cystic fibrosis. Acta Paediatr 2002;91:82-87.

7. Chillon M, Dörk T, Casals T, Giménez J, Fonknechten N, Will K et al. A novel donor splice site in intron 11 of the CFTR gene, created by mutation 1811+1.6kbA>G, produces a new exon: high frequency in Spanish Cystic Fibrosis chromosomes and association with severe phenotype. Am J Hum Genet 1995;56:623-629.
